# Robust in-vehicle heartbeat detection using multimodal signal fusion

**DOI:** 10.1038/s41598-023-47484-z

**Published:** 2023-11-27

**Authors:** Joana M. Warnecke, Joan Lasenby, Thomas M. Deserno

**Affiliations:** 1 Peter L. Reichertz Institute for Medical Informatics of TU Braunschweig and Hannover Medical School, 38106 Brunswick, Germany; 2 Department of Engineering, University of Cambridge, Cambridge, CB2 1PZ UK

**Keywords:** Biomedical engineering, Lifestyle modification

## Abstract

A medical check-up during driving enables the early detection of diseases. Heartbeat irregularities indicate possible cardiovascular diseases, which can be determined with continuous health monitoring. Therefore, we develop a redundant sensor system based on electrocardiography (ECG) and photoplethysmography (PPG) sensors attached to the steering wheel, a red, green, and blue (RGB) camera behind the steering wheel. For the video, we integrate the face recognition engine SeetaFace to detect landmarks of face segments continuously. Based on the green channel, we derive colour changes and, subsequently, the heartbeat. We record the ECG, PPG, video, and reference ECG with body electrodes of 19 volunteers during different driving scenarios, each lasting 15 min: city, highway, and countryside. We combine early, signal-based late, and sensor-based late fusion with a hybrid convolutional neural network (CNN) and integrated majority voting to deliver the final heartbeats that we compare to the reference ECG. Based on the measured and the reference heartbeat positions, the usable time was 51.75%, 58.62%, and 55.96% for the driving scenarios city, highway, and countryside, respectively, with the hybrid algorithm and combination of ECG and PPG. In conclusion, the findings suggest that approximately half the driving time can be utilised for in-vehicle heartbeat monitoring.

## Introduction

According to the World Health Organization, cardiovascular diseases cause 32% of global deaths, which are 17.9 million deaths per year^1^. Stroke and heart attacks are responsible for 85% of these deaths^1^. Atrial fibrillation is a risk factor for both stroke^2^ and heart failure^3^, and often yields an abnormally fast and irregular heartbeat^4^. Continuous monitoring of heartbeats enables early detection, improves therapeutic outcomes, and decreases the mortality rate^5^. In Western countries, people spend about 35 min per day driving a vehicle^6^. This time could be used for a medical check-up – without any additional burden on behaviour change or time – and continuous monitoring can be integrated into our daily life^7^. So far, commercial in-vehicle systems for health-related monitoring focus on the tiredness tracking of the driver. For instance, they track the eyes and movements of the steering wheel or the pedals, as well as car-to-lane distances^8–10^.

However, it is also possible to monitor individual health in a medical sense^7^. Several publications have focused on heartbeat detection during driving^11–14^. In our previous work, we conducted a review to identify relevant sensors for in-vehicle health monitoring^15^. In 2010, Vavrinsky et al. integrated one-lead electrocardiogram (ECG), galvanic skin response, and temperature sensors into each side of the steering wheel^12^. In the same year, Lazaro et al. applied radar from the seat backrest for heart and respiratory rate detection^13^. In 2011, Walter et al. integrated capacitive ECG and ballistocardiography (BCG) into the seat belt^14^. In 2012, Gomez-Clapers & Casanella attached electrodes for ECG to the steering wheel to derive the heart rate^16^. In 2015, Kuo et al. introduced image-based photoplethysmography (iPPG) for drivers’ heart rate detection^17^. More recently, we developed ECG electrodes integrated into the steering wheel^18^. We replaced the previous copper electrodes^19^ with printed and flexible electrodes to improve the SNR. To prevent any impact on driving behavior, our flexible and thin polyurethane electrodes exactly fit the three-dimensional (3D) shape of the steering wheel^18^. However, the quality of health monitoring based on a single sensor is insufficient^11^. Redundant sensor systems have been used in aerospace^20^ and autonomous driving^21^. They can guard against the fact that one defective sensor may yield wrong assumptions and could cause severe adverse events.

In a redundant sensor system, a fusion of the sensor data is needed. Münzner et al.^22^ compared three convolutional neural network (CNN)-based approaches: early, signal-based late, and sensor-based late fusion. The early fusion merges data in the convolutional layer. This layer extracts important signal features. Sensor-based late fusion merges the input data in the dense layer. The dense layer classifies the signal into binary classes. The signal-based late fusion includes two CNNs per signal, which increases the number of features and the computing time. Tejedor et al. reviewed signal fusion in the biomedical domain, in particular, for reliable heart rate detection^23^. They highlight the CNN-based information fusion from Chandra et al.^24^. This algorithm can be applied to noisy data. Furthermore, we focused on the development of a redundant sensor system in a driving simulator, which was composed of ECG, PPG, BCG, and iPPG sensors^19^.

We use the sensor system as well as the data fusion algorithm. Based on our previous results, we select the sensors with the best performance, which are ECG, PPG, and iPPG. We record the data with 19 subjects under real driving conditions. Furthermore, we develop fusion approaches and determine the performance gain on the reliability of heartbeat detection. Altogether, we want to answer the research questions: (1) *Which driving time is utilisable to detect the heartbeat robustly and accurately in the vehicle?*, (2) *What is the most reliable combination of sensors*, and (3) *How does the heartbeat detection performance vary between different driving scenarios?*.

## Methods

### Ground truth

We obtain the ground truth with an ECG sensor (BiosignalPlux Explorer, Plux Wireless Biosignals, Lisbon, Portugal) connected to three adhesive electrodes, which we attach to the usual positions on the chest^25^. We make a test recording to ensure the electrodes are in the correct position. The R-waves are detected by the *simultaneous truth and performance level estimation* (STAPLE) algorithm from Kashif et al.^26^. The STAPLE algorithm includes nine state-of-the-art algorithms: Pan and Tompkins^27^, Chernenko^28^, Arzeno et al.^29^, Manikandan et al.^30^, Lentini et al.^31^, Sartor et al.^32^, Liu et al.^33^, Arteaga-Falconi et al.^34^, and Khamis et al.^35^. STAPLE determines the positions of the R-waves based on a majority vote. We implement and execute our algorithms using a script-based math package (MATLAB version R2021a, The MathWorks, Natick, United States).

### Experimental design

We record data from $$N=19$$ volunteering subjects driving a vehicle with an automatic gear shift (VW Tiguan 2.0 4M RL, Volkswagen AG, Wolfsburg, Germany). The volunteers from diverse ethnicities differ in gender (female: $$n=6$$ and male: $$n=13$$), age (20-67 years), height (164-195 cm), weight (63-120 kg), having a beard ($$n=4)$$, and wearing glasses ($$n=8$$). For volunteers with long hair ($$n=7$$), the outer segment of the forehead is covered. We record 15 min for each scenario *city*, *highway*, and *countryside* in Braunschweig (Lower Saxony, Germany).

To ensure comparable recordings, all volunteers drove the same route. The *city* requires many start and stop maneuvers. The first part of the *highway* is bumpy. In addition, a construction zone causes traffic jams, and a long tunnel degrades the light for camera recording. The *countryside* route leads along rural roads and smaller villages and has a railroad crossing.

### Sensor system

**Figure 1:**
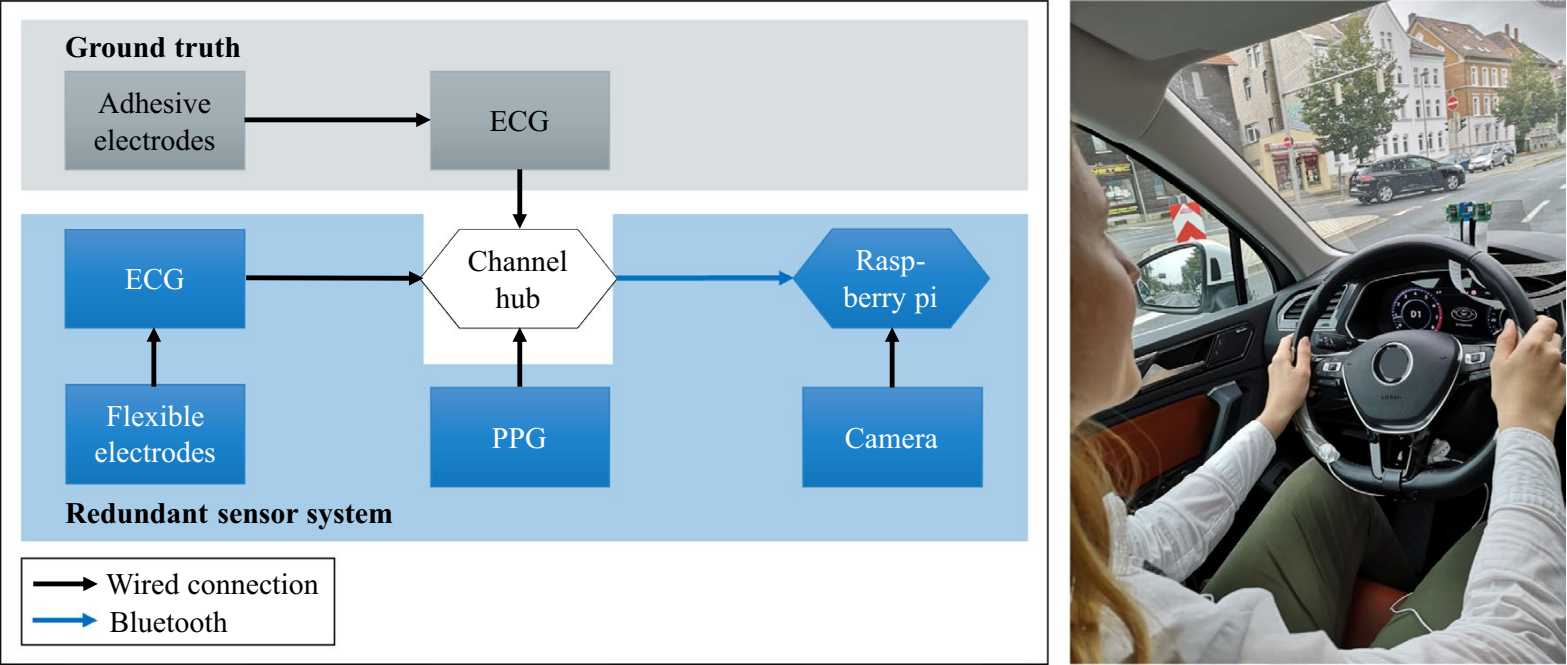
Left-hand side: Schematic diagram of the sensor system. Right-hand side: In-cabin sensor system during the scenario *city*.

**Figure 2:**
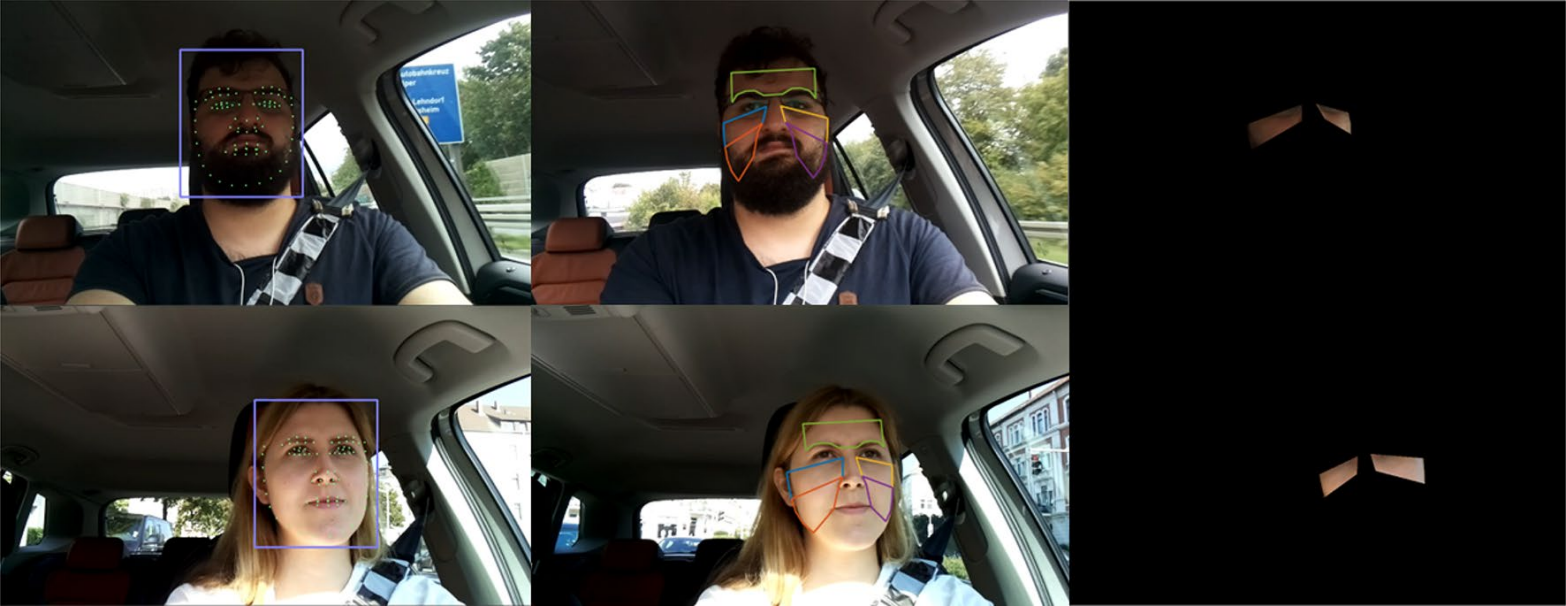
Recording during the driving scenario *highway* (top) and *city* (bottom). Left: Landmark detection with *SeetaFace*. Middle: Applied face segments. Right: Face segments for cheek. Figure 2 is generated with MATLAB (MATLAB version R2021a, The MathWorks, Natick, United States).

We use steering wheel-based contact ECG and PPG sensors and RGB camera for iPPG in front of the driver behind the steering wheel as redundant recording systems. We select an ECG sensor (BiosignalPlux Explorer, Plux Wireless Biosignals, Lisbon, Portugal) and a PPG sensor with two integrated LEDs for the red and infrared spectrum (BiosignalPlux Explorer, Plux Wireless Biosignals, Lisbon, Portugal). We positioned the PPG sensor on the steering wheel’s right side at the level of the index finger. A channel hub (BiosignalPlux Explorer, Plux Wireless Biosignals, Lisbon, Portugal) connects the ECG and PPG sensors and sends the recorded data via Bluetooth to a single-board computer (Raspberry Pi, Raspberry Pi Foundation, Cambridge, United Kingdom). The sampling rate for ECG and PPG is 500 Hz. The red, green, and blue (RGB) camera (Raspberry Pi Foundation, Cambridge, United Kingdom) has a wired connection to the single-board computer (Fig. 1). It records 10 frames per second (FPS) with 720 by 1280 pixels. For time synchronization, we develop a Python script on the single-board computer with an integrated counter that assigns the same ascending number to each sample at the same point in time. The subjects in Figs. 1 and 2 signed an informed consent and agreed to the publication of the image in an online open-access journal.

### Ethics approval

We record all data based on the Helsinki Declaration. The study design was approved by the ethics committee (Internal process number: D_2022-13) of the TU Braunschweig (Lower Saxony, Germany)^36^. Informed consent was obtained from participants.

## Signal pre-processing

### Face recognition and face segmentation

We use the face recognition engine Seetaface (version SeetaFace2^37^) to detect landmarks on the driver’s face. As a first step, it applies a funnel-structured cascade schema for face detection. Second, it cascades several stacked auto-encoder networks for landmark detection and inherits a modified AlexNet for face composition^38^. We extract the cheeks as a region of interest (ROI) based on the landmarks. According to Kamshilin et al.^39^, we extract the green channel and detect color changes caused by the systolic and asystolic blood flow (Fig. 2). We select the right and left cheeks as ROI because more capillaries are in these ROIs compared to the forehead, leading to better results than other regions^40,41^. Moreover, hair and beard, as well as glasses, may cover the skin in the ROI (Fig. 2). Varying illumination further impacts the signal quality^17^.

### Implementation for statistical analysis

We generated the ground truth with the STAPLE algorithm from Kashif et al.^26^ that is implemented in MATLAB (version R2021a, The MathWorks, Natick, United States) and uses the MATLAB Signal Processing Toolbox. We extracted the facial landmarks with SeetaFace2^37^, which uses packages from OpenCV (version 4.5) and CMake (version 3.16). For the fusion approach, we used the libraries TensorFlow (version 2.3.1) and Keras (version 2.4.3) with Python (version 3.8.5) as the programming language. The evaluation is made with Numpy (version 1.19.0).

### Input data

**Figure 3:**
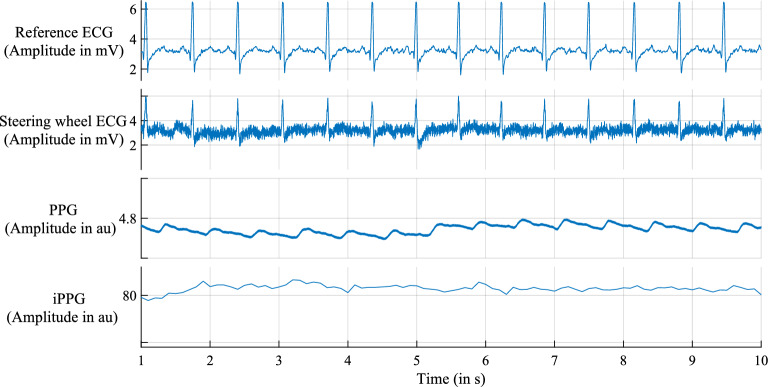
Recorded signals from subject 0001 during the scenario *highway* from second 1 to 10. Figures 3 and 4 are created with MATLAB (MATLAB version R2021a, The MathWorks, Natick, United States).

**Figure 4:**
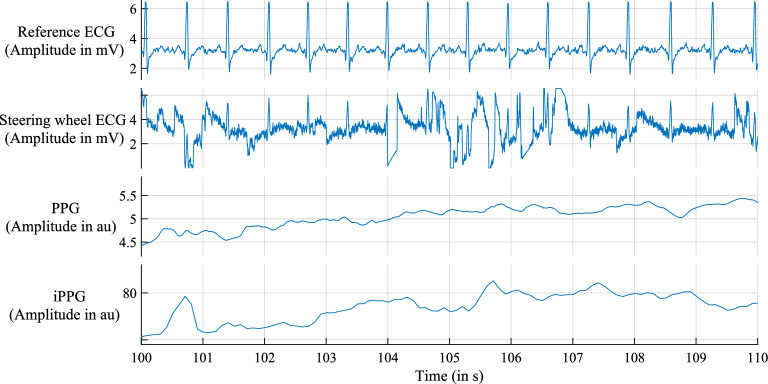
Recorded signals from subject 0001 during the scenario *highway* from second 100 to 110.

The input contains the ECG (steering wheel) and PPG signals, as well as the green channel of the RGB video. According to Chandra et al. ^24^, we (i) up-sampled the data to 500 Hz, (ii) applied median filtering of size 100 samples, and (iii) normalized the amplitudes to the range of $[-1,1]$. The signal quality changes over time due to the movements of the driver and vehicle, which are caused by normal driving activities (Figs. 3, 4). This leads to baseline wander, noise, and artifacts (Fig. 4).

However, the recordings of the reference ECG have a high signal-to-noise ratio (SNR), which is used as a ground truth. The arbitrary unit (au) represents the unit for PPG and iPPG (Figs. 3, 4). As suggested by Chandra et al.^24^, we split the signal into snippets of 501 overlapping samples. The overlap is 490 and 500 for generating training and testing snippets, respectively. To create more training data, the overlap for the training data is reduced. We use leave-one-subject-out cross-validation: one subject is used as a test set, and the remaining as a training set, which is repeated 19 times, and the results are averaged.

## Signal fusion

Our hybrid signal fusion approach has a CNN structure and determines the signal segments containing a heartbeat^19^. The model parameters are matched for driving scenarios. It includes (i) early fusion, (ii) signal-based late fusion, and (iii) sensor-based late fusion. The voting function finally determines the heartbeats’ positions^19^.

**Figure 5:**
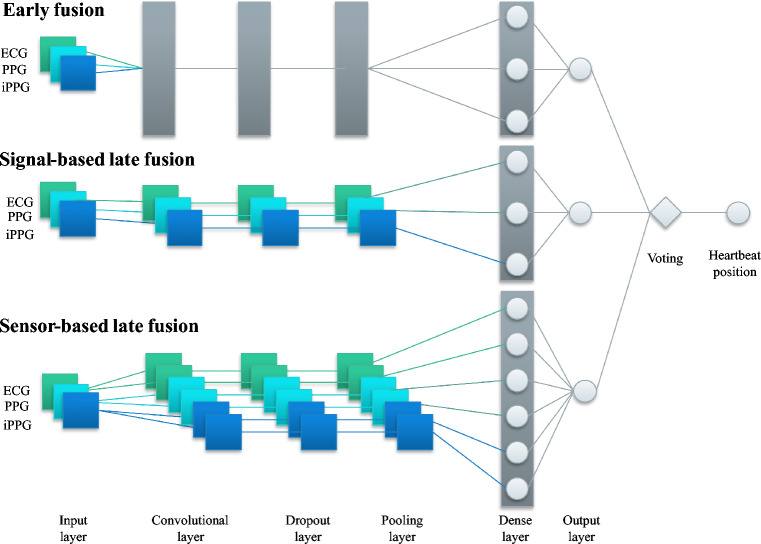
Hybrid fusion approach for three signals.

The input layer is the first layer, and the inputs are ECG, PPG, and iPPG (Figs. 3 and 4). The convolutional layer extracts features from the signals and generates a feature map. In line with Chandra et al.^24^, we use two filters with a kernel size of 20 for each sensor signal in the convolutional layer. To prevent over-fitting, the dropout layer has a dropout rate of 0.5. The pooling layer minimises unnecessary information with the function MaxPooling1D, and the pool size is 2. The dense layer binary classifies snippets: no heartbeat (class 0) and heartbeat (class 1). We choose the sigmoid as an activation function and an Adam optimizer with a learning rate of 0.001. The output layer generates a vector $\hat{Y}$ of multiple labels that are either 0 or 1. The voting function is independent of the CNN and processes $\hat{Y}$ for the final decision based on a majority vote. Thereby, *j* represents the number of a snippet, and *s* stands for the signal. The output vector is^19^:

1 $$\begin{aligned} \hat{Y}=\left\{ \hat{y}_{1 j}+\hat{y}_{2 j}+. .+\hat{y}_{n j}\right\} \forall j=[1, s] \end{aligned}$$

We compute training and testing on the high-performance computer *Phoenix* at TU Braunschweig^42^.

Early fusion and signal-based late fusion have a single integrated CNN (Fig. 5). In the context of signal-based late fusion, both CNNs receive identical input data for each signal. In contrast to sensor-based fusion, it extracts much more parameters.

In the early fusion approach, the convolutional layer extracts features from input signals, such as the R-wave in the ECG or the systolic peak in the PPG. These features are subsequently aggregated within a feature map, which is further processed in a dropout layer. In contrast, sensor-based fusion yields a larger number of extracted parameters in comparison to signal-based late fusion. The visualisation shows the hybrid fusion with the input of three signals. The input layer contains one or two signals for the performance comparison. The voting function operates independently of the CNN and evaluates the output vector $\hat{Y}$ to make the final decision regarding the presence of heartbeats in a given segment. This decision is based on a majority vote, where if more than two sensor fusion approaches yield a label $\hat{Y} = 1$ it is inferred that the segment contains a heartbeat (class 1), else not (class 0)^19^.

## Evaluation

For evaluation, we follow the approach of Chandra et al.^24^. Moreover, we chose this approach because it reflects the relation between false-positive (FP), false-negative (FN), and true-positive (TP).

Accordingly, TP, FN, and FP determine whether an R-wave was correctly detected, missed, or a spurious spike was mistaken for an R-wave. Due to the high number of true negatives, specificity is not used. We calculate an overall performance:

2 $$\begin{aligned} P = \frac{\text{PPV}+\text{S}}{2}, \end{aligned}$$

that uses the positive predictive value

3 $$\begin{aligned} {\text{PPV}} = \frac{\text{TP}}{{\text{TP}}+{\text{FP}}}, \end{aligned}$$

and the sensitivity

4 $$\begin{aligned} {S} = \frac{\text{TP}}{{\text{TP}}+{\text{FN}}}. \end{aligned}$$

We compare *P* between the different signal pairs and also the fusion of all three signals in the three scenarios *city*, *highway*, and *countryside*.

## Results

### Performance of a single signal

The PPG signal achieves the highest performance ($P_{max} = 57.25\%$) with early fusion. This means that 57.25% of the recording time, the heartbeat position matches the ground truth (Table 1). In total, PPG delivers the highest score three times for a specific signal fusion approach. Mean_P_ denotes the mean performance for a signal fusion approach. The signal-based late approach has highest Mean_P_ (Mean_P_ = 50.54%).

**Table 1:** Performance of one signal for all scenarios.

Approach	ECG (%)	PPG (%)	iPPG (%)	Mean_P_ (%)
Early Fusion	44.33	**57.25**	48.50	50.02
Signal-based late fusion	49.98	**51.27**	50.38	**50.54**
Sensor-based late fusion	**50.10**	49.41	45.35	48.29
Hybrid algorithm	48.10	**52.09**	47.71	49.30

Significant values are in [bold].

### Performance of two signals

The early fusion of PPG+iPPG has the highest performance $P=55.79\%$ (Table 2). Overall scenarios, the tables show that the ECG+PPG combination achieves the highest performance two times. In comparison, the sensor pair ECG+iPPG and PPG+iPPG only achieves the highest score once. The hybrid algorithm has the highest Mean_P_ (Mean_P_ = 52.61%).

**Table 2:** Performance of two signals for all scenarios.

Approach	ECG+PPG (%)	ECG+iPPG (%)	PPG+iPPG (%)	Mean_P_ (%)
Early Fusion	54.09	47.36	**55.79**	52.41
Signal-based late fusion	**53.43**	52.89	48.13	51.48
Sensor-based late fusion	49.95	**50.90**	50.33	50.40
Hybrid algorithm	**55.44**	51.82	50.57	**52.61**

Significant values are in [bold].

### Performance of three signals

The sensor-based late fusion approach achieves the highest performance twice with the scenarios *city* ($P=57.23\%$) and *highway* ($P=57.50\%$) (Table 3). The signal-based late fusion has the highest performance for the *countryside* ($P=47.16\%$). The sensor-based late fusion approach has the second-ranked performance in the *countryside* ($P=42.00\%$). Therefore, this approach yields the best performance. With the sensor-based late fusion approach, the correct heartbeat is detected on average for 52.24% of the driving time. Early fusion yields the lowest score twice: *city* ($P=47.90\%$) and *highway* ($P=48.52\%$). The scenario *highway* has the highest score ($P=57.50\%$), followed by *city* ($P=57.23\%$) and *countryside* ($P=47.16\%$).

**Table 3:** Performance of three signals during driving.

Approach	City (%)	Highway (%)	Countryside (%)	Mean_P_ (%)
Early fusion (ECG+PPG+iPPG)	47.90	48.52	36.64	44.35
Signal-based late fusion (ECG+PPG+iPPG)	53.41	51.57	**47.16**	50.71
Sensor-based late fusion (ECG+PPG+iPPG)	**57.23**	**57.50**	42.00	**52.24**
Hybrid algorithm (ECG+PPG+iPPG)	49.49	54.62	33.95	46.02

Significant values are in [bold].

### Best performance with ECG and PPG and hybrid fusion

**Figure 6:**
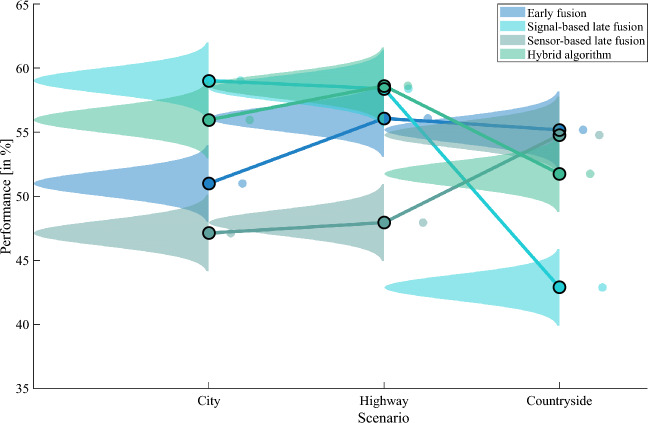
Performance of ECG+PPG for different driving scenarios. Figure 6 is based on the MATLAB package from Allen et al.^43^ (MATLAB version R2021a, The MathWorks, Natick, United States).

The distribution plot shows the performance differences during the different driving scenarios (Fig. 6). The hybrid fusion approach delivers, on average, the best performance with the ECG and PPG sensor ($Mean_{P}=55.44\%$) (Table 4). The sensor-based late fusion and early fusion have the lowest performance (Table 4). This is 3.2% higher than with the sensor-based late fusion with ECG+PPG+iPPG.

**Table 4:** Performance overview of ECG and PPG.

Approach	City (%)	Highway (%)	Countryside (%)	Mean_P_ (%)
Early Fusion (ECG+PPG)	55.18	56.08	51.00	50.02
Signal-based late fusion (ECG+PPG)	42.89	58.39	59.02	53.43
Sensor-based late fusion (ECG+PPG)	54.78	47.95	47.13	49.95
Hybrid algorithm (ECG+PPG)	51.75	58.62	55.96	**55.44**

Significant values are in [bold].

## Discussion

A big challenge of continuous in-vehicle health monitoring is the poor signal quality due to the movements of the car and the driver^14^, (partly abrupt) changes in the illumination^44^, characteristics such as skin color^45^, hair and beard cuts, and physiological parameters: humans with a lower R-wave amplitude yield lower performance. Our sensor system is composed of ECG, PPG, and iPPG on and behind the steering wheel. For ECG, we printed polyurethane electrodes exactly in the 3D shape of the steering wheel^18^. Comparing the sensor pairs identifies the best-performing sensor, and the selection of sensor pairs increases the resource efficiency.

The novelty of our work is the data collection and analysis of biomedical data under real driving conditions. Moreover, we use a multimodal fusion method, which is already applied to other use cases, such as wearable IoT sensors^46^. Previous publications for in-vehicle health monitoring mostly focus on the analysis of a single sensor^11,14,17^. For instance, in 2018, Leicht et al. evaluated the capacitive ECG in a driving simulator^47^. Although Walter et al. measured the cECG and BCG during driving, they reported the technical implementation rather than the fusion of signals or the portion of usable driving time^14^. Contrarily, we compared the R peak positions between the ground truth and the steering wheel ECG, and we determined the correct heartbeat in 45.62% of the driving time^18^. However, the hybrid signal fusion of three signals outperforms our previous results for heartbeat detection under real driving conditions.

Furthermore, we integrated face recognition based on 81 landmarks, which changes with every movement, to detect the face segments. The performance of the iPPG is worse than the performance of the ECG and PPG sensors. This leads to a better performance of two instead of three sensors. However, a redundant system still improves performance and enables continuous monitoring during driving.

The current sensor system can still be improved by additional PPG sensors, which are placed on the steering wheel. Additional PPG sensors could be placed around the steering wheel to increase the possibility of recording a signal with a good SNR. The recording of the iPPG signal is often disturbed by strong sunshine. Also, it is possible to optimize the structure of the signal fusion model. Further research could investigate the difference with respect to the performance with adjusted model parameters by increasing the number of convolutional layers, for instance^48^. Furthermore, a lower or varying learning rate of the Adam optimizer could improve the results^49^. There is also an option to use another activation function, e.g., rectified linear unit (ReLU) or leaky ReLU function^50^.

Majority voting and averaging are two common techniques used in signal fusion, and their effectiveness depends on the specific context and characteristics of the signals being fused. We selected majority voting due to its robustness against outliers and noisy data points, which are less likely to influence the final decision as the majority overrules them. Majority voting is especially valuable in situations where discrete or categorical decisions are required, such as in classification tasks or binary decision-making.

The redundant system also has some limitations. We excluded the BCG sensor because the pretest in the car showed that the SNR was low. For the pre-test, we placed the sensor at the backrest to measure ballistic forces generated by the heart. Additionally, more testers are needed with different skin colors and melanin levels because these factors have an impact on video-based heartbeat detection^51^. Another important integration is the detection of respiratory rate and temperature. This would enable the detection of a wider variety of diseases. For future work, we will integrate movement detection using depth cameras, as suggested by Fu et al.^52^. Furthermore, the redundant system can be extended by phonocardiography, which records acoustic signals during a cardiac cycle. Such sensors may be integrated into the seat belt to rest on top of the heart.

To detect heartbeat arrhythmia, it is important to analyse a longer signal segment with a high SNR. The signal visualisation in Fig. 3 shows that the recorded signal contains such signal lengths. The number of longer segments with a high SNR will be lower than predicted, with approximately 55% of usable driving time. To identify such longer segments, an artifact index^53^ is needed, which includes CAN-BUS data^54^, such as the acceleration of the steering wheel and the car.

We will publish a research paper focusing on respiratory rate detection during driving, an essential vital sign for monitoring the driver’s health and detecting respiratory diseases. The study involves 15 healthy subjects and follows the same experimental design as this paper^55^. We present our findings in two separate papers due to the complexity of the sensor system, pre-processing, and CNN-based training parameters, which exceed a single journal paper’s scope.

## Conclusion

In summary, we developed a redundant sensor system and signal fusion approaches to detect heartbeats while driving. Moreover, we want to answer the research question of the usable driving time for heartbeat detection: The hybrid algorithm and sensor pair ECG and PPG deliver on average the best results *highway* ($P=58.62\%$), *countryside* ($P=55.96\%$), and *city* ($P=51.75\%$).

As a take-home message, we can potentially use over half of our drive time for continuous monitoring with the ECG and PPG sensor and a low variance between the different driving scenarios. This provides the possibility to detect symptoms of cardiovascular diseases at an earlier stage in comparison to conventional methods. With the publicly available data, it is possible to reproduce the results and apply further algorithms to detect the correct heartbeat position.

## Data Availability

Due to the inability to derive the identity of the subject from the ECG, PPG, and iPPG signals, we published the data anonymously in the TU Braunschweig library under CC BY 4.0 (link: https://doi.org/10.24355/dbbs.084-202207150657-0, accessed on November 16th, 2023). The dataset contains reference ECG, steering wheel ECG, and PPG (.csv format), RGB channels for face segment cheek (.mat format), and subject information (subject ID, age, height, weight, gender, and known diseases). All test persons signed a consent form and agreed on the publication of these anonymous data.
