# Author Correction: Robust in-vehicle heartbeat detection using multimodal signal fusion

**DOI:** 10.1038/s41598-024-56883-9

**Published:** 2024-03-14

**Authors:** Joana M. Warnecke, Joan Lasenby, Thomas M. Deserno

**Affiliations:** 1 Peter L. Reichertz Institute for Medical Informatics of TU Braunschweig and Hannover Medical School, 38106 Brunswick, Germany; 2 Department of Engineering, University of Cambridge, Cambridge, CB2 1PZ UK

Correction to: *Scientific Reports* 10.1038/s41598-023-47484-z, published online 27 November 2023

The original version of this Article contained an error in Reference 55, which was incorrectly given as:

Warnecke, J.M., Lasenby, J. & Deserno, T.M. Robust in-vehicle respiratory rate detection using multimodal signal fusion https://doi.org/10.21203/rs.3.rs-2828298/v1 (2023).

The correct reference is listed below:

Warnecke, J.M., Lasenby, J. & Deserno, T.M. Robust in-vehicle respiratory rate detection using multimodal signal fusion. Sci Rep 13, 20435. https://doi.org/10.1038/s41598-023-47504-y (2023).

The original Article has been corrected.

